# Synergistic Effects of AgNPs and Biochar: A Potential Combination for Combating Lung Cancer and Pathogenic Bacteria

**DOI:** 10.3390/molecules28124757

**Published:** 2023-06-14

**Authors:** Maha N. Abu Hajleh, Muhamad Al-limoun, Amjad Al-Tarawneh, Tahani J. Hijazin, Moath Alqaraleh, Khaled Khleifat, Osama Y. Al-Madanat, Yaseen Al Qaisi, Ahmad AlSarayreh, Ali Al-Samydai, Haitham Qaralleh, Emad A. S. Al-Dujaili

**Affiliations:** 1 Department of Cosmetic Science, Pharmacological and Diagnostic Research Centre, Faculty of Allied Medical Sciences, Al-Ahliyya Amman University, Amman 19328, Jordan; m.abuhajleh@ammanu.edu.jo; 2 Department of Biological Sciences, Faculty of Science, Mutah University, P.O. Box 7, Mutah 61710, Jordan; moallimoun@mutah.edu.jo (M.A.-l.); thijazeen@yahoo.com (T.J.H.); yastq@yahoo.com (Y.A.Q.); ahmsar@mutah.edu.jo (A.A.); 3 Prince Faisal Center for Dead Sea, Environmental and Energy Research, Mutah University, Al-Karak 61710, Jordan; amjtar@hotmail.com; 4 Pharmacological and Diagnostic Research Center (PDRC), Faculty of Pharmacy, Al-Ahliyya Amman University, Amman 19328, Jordan; alqaralehmoath@yahoo.com (M.A.); a.alsamydai@ammanu.edu.jo (A.A.-S.); 5 Department of Medical Analysis, Faculty of Science, Mutah University, Al-Karak 61710, Jordan; haitham@mutah.edu.jo; 6 Department of Chemistry, Faculty of Science, Mutah University, Al-Karak 61710, Jordan; madanat@mutah.edu.jo; 7 Centre for Cardiovascular Science, Queen’s Medical Research Institute, University of Edinburgh, Edinburgh EH8 9YL, UK

**Keywords:** synergism, biochar, AgNPs, anticancer, anti-inflammatory, antibacterial

## Abstract

The synthesis of reliable biological nanomaterials is a crucial area of study in nanotechnology. In this study, Emericella dentata was employed for the biosynthesis of AgNPs, which were then combined with synthesized biochar, a porous structure created through biomass pyrolysis. The synergistic effects of AgNPs and biochar were evaluated through the assessment of pro-inflammatory cytokines, anti-apoptotic gene expression, and antibacterial activity. Solid biosynthesized AgNPs were evaluated by XRD and STEM, with STEM images revealing that most of the AgNPs ranged from 10 to 80 nm, with over 70% being less than 40 nm. FTIR analysis indicated the presence of stabilizing and reducing functional groups in the AgNPs. The nanoemulsion’s zeta potential, hydrodynamic diameter, and particle distribution index were found to be −19.6 mV, 37.62 nm, and 0.231, respectively. Biochar, on the other hand, did not have any antibacterial effects on the tested bacterial species. However, when combined with AgNPs, its antibacterial efficacy against all bacterial species was significantly enhanced. Furthermore, the combined material significantly reduced the expression of anti-apoptotic genes and pro-inflammatory cytokines compared to individual treatments. This study suggests that low-dose AgNPs coupled with biochar could be a more effective method to combat lung cancer epithelial cells and pathogenic bacteria compared to either substance alone.

## 1. Introduction

Nanoscience, one of the most prominent areas of modern research, explores the composition, structure, and properties of nanoparticles. Since nanotechnologies are applied in several other disciplines, such as engineering [1], medicine, pharmacy, environmental remediation [2], and sustainable fuel production [3,4], they are recognized as one of the most current and active study areas [5,6]. The last ten years have seen a tremendous global expansion of the vast multidisciplinary field of nanotechnology [7,8]. Nanomaterials that have been created or modified using biotechnology are the focus of nanotechnology [9]. Fungal processes are used to create nanoparticles of metals, which have a wide range of uses such as materials, textiles, in food preservation, disease detection and control, and wound healing [10]. Other physical and chemical processes for creating nanoparticles (NPs) use hazardous substances and are expensive [11]. Due to their nanotoxicity, the biological method is currently used as a temporary option because it poses fewer environmental and health risks [12,13]. Therefore, environmentally friendly, straightforward, and efficient methods have been adopted for NP biosynthesis, which is crucial due to their lower toxicity and benign ecological impact. The behavior, biodistribution, effectiveness, and safety of nanoparticles are influenced by their physicochemical characteristics. To assess the functional qualities of the produced particles, the characterization of NPs is important. Various techniques can be used, including atomic force microscopy (AFM), scanning electron microscopy (SEM), transmission electron microscopy (TEM), and X-ray photoelectron spectroscopy (XPS) [14,15]. Silver nanoparticles (AgNPs) are primarily employed in medicine for their therapeutic and diagnostic applications in addition to their antimicrobial properties. Due to their unique physical, chemical, and optical properties, silver nanoparticles are attracting growing interest [16]. Antibacterial and anticancer activities are already present in silver nanoparticles [17,18]. It may be possible to treat cancers that stop responding to chemotherapy or radiotherapy by combining AgNPs’ natural anticancer properties with anticancer pharmaceuticals, or by using these AgNPs as concurrent target-directed drug delivery platforms for anticancer drugs [19].

Biochar, a pyrolyzed material with a high carbon content can be made from a range of organic materials [20,21]. Biochar has a porous structure that enables it to hold and collect small molecules including nanoparticles [22]. Combining biochar with AgNPs may boost both compounds’ potential antibacterial properties by lowering the doses of harmful bioactive molecules [23]. Recent findings have shown that biochar reduces the bioavailability of AgNPs through the complexion of surfaces, inhibiting their solubility capacity, and releasing damaging Ag+ ions into the growth media [24]. Previously, similar results were reported indicating that the presence of biochar caused tissue Ag levels to drop by at least a factor of two and efficiently prevent their bioaccumulation [25]. In addition, there have been few reports of the potential health risks associated with utilizing biochar. Further investigations are required to determine whether biochar can be used to boost the activity of silver nanoparticles against cancer cell lines and diverse microbial infections [26]. The aim of this study was to biosynthesize novel fungal-mediated AgNPs, describe their characteristics, and investigate AgNPs’ antibacterial, anti-inflammatory, and anticancer properties using the A549 cell line, either alone or in combination with biochar.

## 2. Results

### 2.1. Silver Nanoparticles (AgNPs) and Biochar

**Figure 1:**
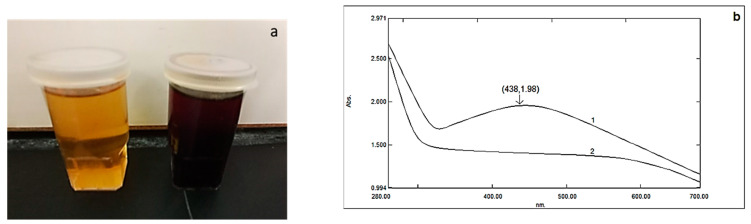
Bio-reduced 1.0 mM AgNO_3_ by Emericella dentata at 35 °C: (**a**) color change and (**b**) UV–visible spectra. Line 1 represents the spectrum for the filtrate while line 2 shows the spectrum for the AgNP suspension.

Although there has been a lot of interest in AgNP biosynthesis, to our knowledge, this is the first report employing the fungus isolate *Emericella dentata* to produce AgNPs. The production of AgNPs was optimized at 10 g biomass and 1.0 mM AgNO_3_ at 35 °C and pH 7.0 for 96 h. The test flask developed a brown hue because of the bioreduction of AgNO_3_ salt via the prepared mycelia filtrate, which did not happen in the control experiment (Figure 1a). A silver nanoparticle SPR peak is visible in Figure 1b UV–vis spectrum at 438 nm.

The study of date seed (DS) biochar produced at a temperature of 550 °C for pyrolysis revealed a pH of 7.9, an organic matter of 98.56%, fixed carbon of 60.2%, a porosity of 73%, a biochar yield of 25%, and water holding capacity (WHC) of 59.7% (Table 1).

**Figure 2:**
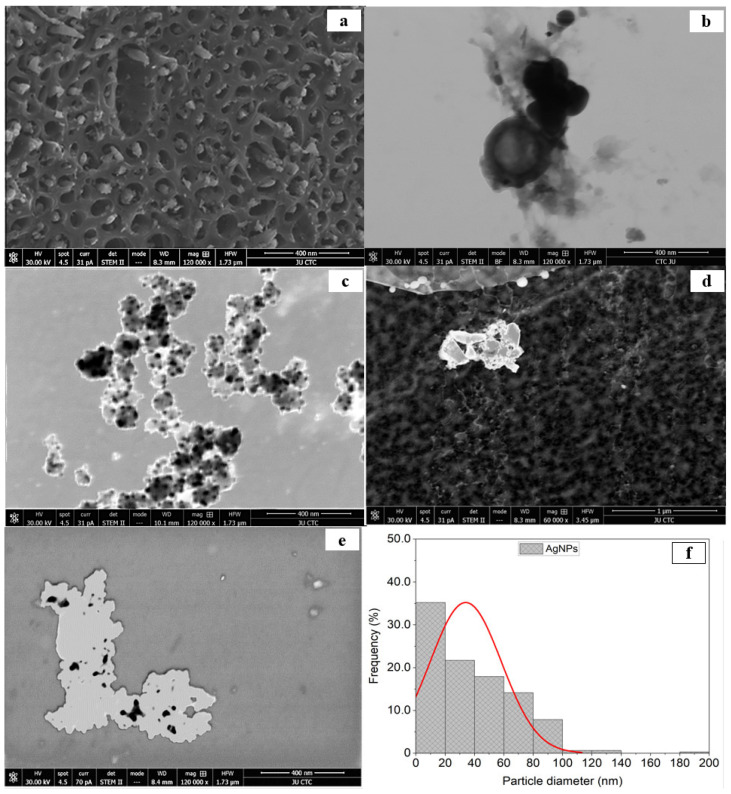
(**a**) STEM micrographs of 400 nm images of biochar, (**b**) STEM micrographs of 400 nm images of biochar [27], (**c**) 400 nm image of silver nanoparticles synthesized by the reaction of 1.0 mM silver nitrate with Emericella dentata filtrate, (**d**) 1000 nm image of combined biochar and AgNPs, (**e**) 400 nm combination of AgNPs and biochar, and (**f**) % frequency of AgNPs size distribution. (Figure 2b shows the identical detail as Figure 2a in [27] and is shown here for easier comparison).

A STEM image (Figure 2a,b [27]) shows the porous surface of used biochar, and the nanoparticles’ STEM image (Figure 2c) shows AgNPs as spherical objects with a size range from 10 to 80 nm. The STEM image of biochar combined with nanoparticles was distorted due to sample charge, the existence of nonconducting carbon stabilizer, and nanoparticle aggregates form bigger composites (Figure 2d); it was fuzzy at higher magnification, making it challenging to determine the structure of the observed AgNPs. STEM micrographs demonstrate that AgNPs decreased in size throughout the drying process, making them smaller than those detected by DLS analysis, where >90% of the AgNPs depicted were smaller than 80 nm, and >70% of the latter were about 40 nm or less (Figure 2e,f).

### 2.2. Particle Size and Zeta Potential

**Figure 3:**
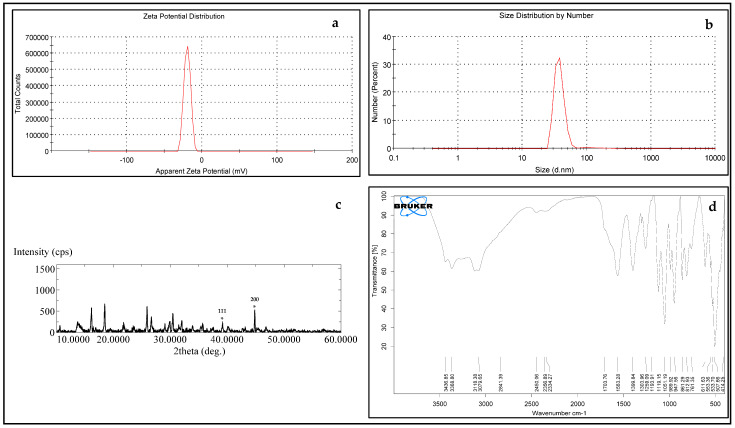
(**a**) Zeta potential distribution of silver nanoparticles [27]; (**b**) the size distribution by the intensity of AgNPs [27]; (**c**) XRD analysis of silver nanoparticles biologically prepared by Emericella dentata filtrate (* assign the reflection planes of the face-centered cubic structure of silver) [27]; and (**d**) ATR-IR of silver nanoparticles biologically prepared by Emericella dentata filtrate. (Figure 3a–c were taken from Figures 1A,B, and 3 in our previous published work [27] and are repeated here for easier comparison).

AgNPs were found to have a particle distribution index of 0.231 and a zeta potential of −19.6 mV, both of which indicate that they are hydrodynamically stable. Their hydrodynamic size diameter averaged up to 45 nanometers. (Figure 3a). The zeta values used to describe the nanoparticle size distribution revealed that 97.3% of the AgNPs were 37.62 nm in size (Figure 3b).

### 2.3. XRD Spectra Analysis

The crystalline nature of the biosynthesized Ag nanoparticles was confirmed by X-ray diffraction analysis (Figure 3c). Two distinct diffraction peaks at 2θ values of 38.1° and 44.1° (assigned with stars) can be assigned to the (1 1 1) and (2 0 0) planes, respectively. The appearance of these patterns indicates that the formation of silver nanoparticles are face-centered cubic structures (fcc) and crystalline in nature (JCPDS file no. 84-0713 and 04-0783) [28]. The appearance of several unassigned peaks observed in the XRD spectrum could be related to the crystallization of the bioorganic compounds from fungi during the preparation of the AgNPs, which further confirms the formation of ligand interaction between the silver nanoparticles and the bio-organic compounds from the fungi extract.

### 2.4. ATR-IR Spectra Analysis

The ATR-IR spectra of silver nanoparticles produced biologically (Figure 3d) show numerous distinct peaks at 633, 989, 1110, 1663, 1704, and 2450, which demonstrated the presence of several organic functional groups that act as reducing and stabilizing agents on the surfaces of silver nanoparticles. The analysis of this spectrum showed a broad band from 3080 to 3437 cm^−1^ assigned to the aromatic amines O–H stretching and the ~N–H stretching vibrations of amide. The presence of the main amine group of proteins is shown by the brevity of the broad peak at 2450 cm^−1^. The appearance of several bands in the regions between 600 and 900 cm^−1^ corresponds to the C-H aromatic out of the plane. The band at 1259 cm^−1^ could be related to the formation of C-O-C stretching in aromatic rings. The strong wide peak between 1400 and 1450 cm^−1^ corresponds to C-H stretching, while the weak band at around 1520 cm^−1^ represents the formation of the C=C aromatic. Bands in the range of 2900–2990 cm^−1^ correspond to C-H stretching. The presence of the amide in the protein’s C=O stretching vibration is indicated by the peaks at 1704, 1663, and 1110 cm^−1^. The stretching frequencies of the amino and amino-methyl stretching groups of proteins have obvious peaks around 1340 cm^−1^. These organizations may oversee the synthesis and conformation of the biological system that accompanies the silver nanoparticles.

### 2.5. Antimicrobial Activity of Silver Nanoparticles

It might be possible that antibiotic resistance could be slowed down by employing innovative approaches, such as the interaction between perfectly treated biochar and silver nanoparticles (AgNPs). The disc diffusion method and the microdilution procedures were used to assess the potential interactions between AgNPs and biochar (Table 2).

The diameters of the inhibitory zones detected by AgNPs alone against *B. cereus*, *L. monocytogene*, *S. epidermidis*, *S. aureus*, *E. coli*, *P. aeruginosa* and *P. aeruginosa* ATCC 10145, *E. coli* 0157:H7, and *E. coli* ATCC 25922 are shown in Table 2. However, none of the bacteria tested were inhibited by the biochar when used alone. The combination of silver nanoparticles (AgNPs) and biochar resulted in a significant increase (*p* < 0.05) in the antibacterial activity of AgNPs against all types of bacteria. When biochar and AgNPs were combined in a ratio of 1:1, the resulting inhibitory zones had the following dimensions: 24.5, 20.5, 14.5, 19.5, 21.5, 16.5, 16.5, 15.0, 19.5, and 17.0 mm for all the microorganisms listed in Table 2. The results of the minimal inhibitory concentration (MIC) were matched by parallel results from the inhibition zone. All the bacteria employed had a MIC when using AgNPS alone ranging between 6.38 and 19.15 (µg/mL). However, the combined treatment of AgNPs and biochar reduced the MIC values to start from 2.13 µg/mL for *B. cereus*, *L. monocytogene*, *S. epidermidis,* and *S. aureus*, and to 6.38 µg/mL for the other investigated bacteria, with a definite reduction in MIC of up to 70% in each case.

### 2.6. Cytotoxicity Effect of AgNPs and Biochar on A549 Cells

**Figure 4:**
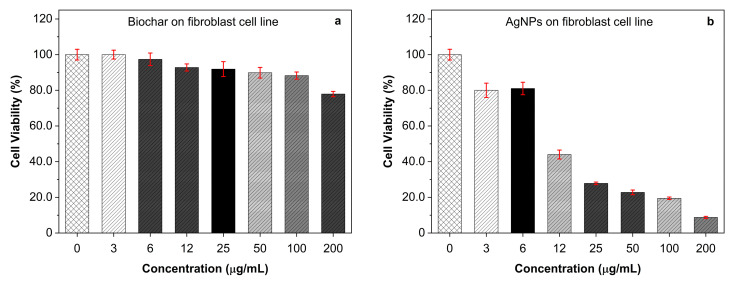
The antiproliferative activity of (**a**) increasing concentrations of biochar and (**b**) increasing concentrations of AgNPs on fibroblast cell line evaluated by MTT assay. The results represent the percentage of cell viability under different concentrations of biochar and AgNPs. All results are expressed as the mean of the three measurements ± SD (n = 3–4 independent replicates).

**Figure 5:**
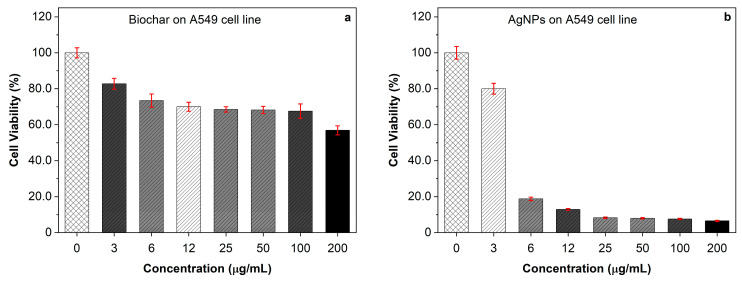
The antiproliferative activity of (**a**) increasing concentrations of biochar and (**b**) increasing concentrations of AgNPs on the A549 cell line evaluated by MTT assay. The results represent the percentage of cell viability under different concentrations of biochar and AgNPs. All results are expressed as the mean of the three measurements ± SD (n = 3–4 independent replicates).

**Figure 6:**
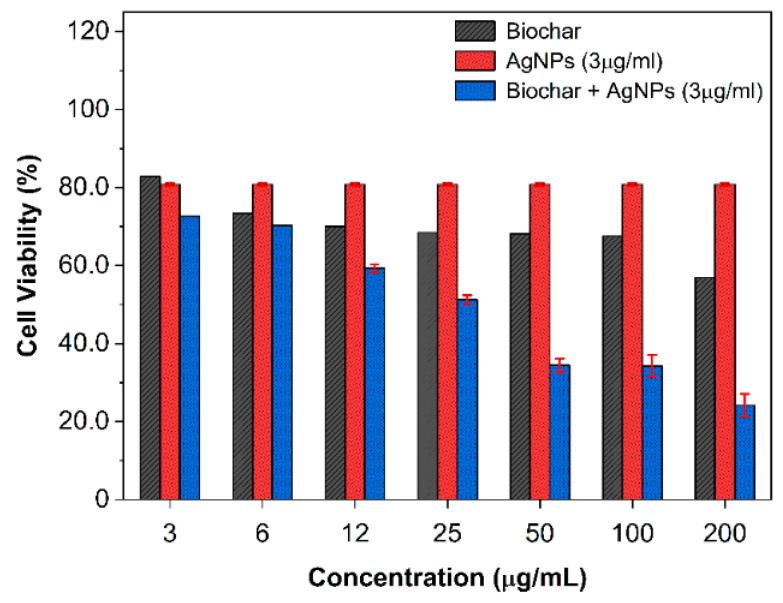
The antiproliferative activity of combined 3 µg/mL AgNPs and increasing concentrations of biochar on the A549 cell line evaluated by MTT assay. The results show cell viability at various biochar concentrations and 3 μg/mL AgNPs. All results are expressed as the mean of the three measurements ± SD (n = 3–4 independent replicates).

The antiproliferative effects of biochar, AgNPs, and their interactions were investigated on fibroblast and A549 (ATCC CCL-185) cell lines. At dosages of 3 and 6 μg/mL, the antiproliferative effects of biochar on human periodontal ligament fibroblast cell lines (PDL) were negligible. However, at doses of 12 μg/mL or higher, there was a discernible inhibitory effect of up to 20% at a concentration of 200 μg/mL (Figure 4a). On the other hand, silver nanoparticles were able to suppress fibroblast cell lines’ (PDL) viability at doses ranging from 12 to 200 µg/mL (Figure 4b). The manner of inhibition was concentration dependent. The cytotoxic effect of biochar on the A549 cell line is demonstrated in Figure 5a, which shows a maximum 40% inhibition of the A549 cell line by biochar at a concentration of 200 µg/mL. When AgNPs were tested against the A549 cell line (Figure 5b), a substantial level of cytotoxicity was seen at doses ranging from 6 to 200 µg/mL, which resulted an in inhibitory effect of up to 90% at the highest concentration tested. For comparison, untreated cells were used as a control. As a result, a constant concentration of 3 µg/mL AgNPs was chosen for future synergy testing against the A549 cell line. The 3 μg/mL dose of AgNPs and various amounts of biochar were applied to the A549 cell line, and the results revealed that the combination was more effective than utilizing each one of them separately (Figure 6). These findings demonstrate that lower AgNP concentrations produced a reasonable level of cytotoxicity and that biochar and AgNPs interact differently when used in combination compared to the use of either agent separately.

### 2.7. Effect of AgNPs and Biochar on the Expression of Gene Regulation

#### 2.7.1. Pro- and Anti-Apoptotic Genes in A549 Cells

**Figure 7:**
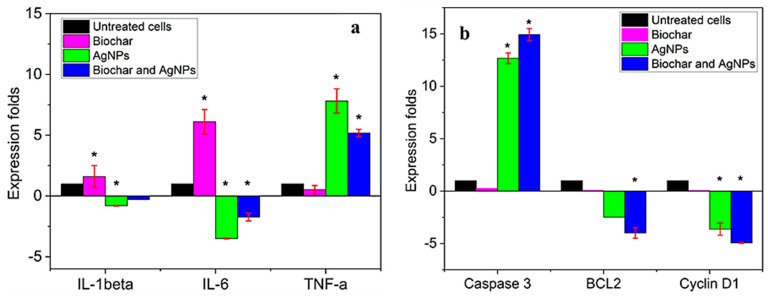
Effect of AgNPs and biochar on the (**a**) expression of caspase 3, BCL-2, and Cylin D1 in the A549 cell line (cells were treated with biochar using the IC50 value and 3 µg/mL of AgNPs) (**b**) the expression of IL-1beta, IL-6, and TNF alpha in the A549 cell line (cells were treated with biochar using the IC50 value and 3 µg/mL of AgNPs). All results are expressed as means of three dependent replicates ± SD. *: *p* < 0.05, compared to control untreated cells.

AgNPs, biochar, and their combinations were studied for their synergistic effects on the expression of pro- and anti-apoptotic genes in A549 cells (Figure 7b). AgNPs (3 µg/mL), biochar (50 µg/mL), or both combined (AgNPs (3 µg/mL) plus biochar (50 µg/mL)) were applied to A549 cells for 24 h. Whilst the IC50 value of biochar alone exerted no significant influence on the expression of caspase 3 and anti-apoptotic genes, including BCL-2 and cyclin D1, the effect of 3 µg/mL of AgNPs alone and biochar with AgNPs produced a statistically significant (*p* < 0.05) upregulation of caspase 3 (the pro-apoptotic genotype) in the A549 cell line compared to untreated cells. However, there was a downregulation of anti-apoptotic genes, including BCL-2 and cyclin D1. In comparison to a single treatment, the combination of AgNPs and biochar significantly reduced the expression of anti-apoptotic genes severalfold. AgNPs combined with biochar trigger apoptosis in A549 lung cancer cells, as demonstrated by our experiments using caspase 3.

#### 2.7.2. The Expression of IL-1β, IL-6, and TNF-α in the A549 Cell Line

Combined treatment with AgNPs and biochar decreased IL-1β and IL-6 levels while raising TNF-α production (Figure 7a). Pro-inflammatory cytokines, IL-1β and IL-6, were negatively regulated in the A549 cell line when exposed to AgNPs (3 µg/mL), and a combination of biochar (50 µg/mL) and AgNPs (3 µg/mL) in comparison to untreated cells (Figure 7b). On the other hand, the IC50 value of biochar alone had an upregulating impact on IL-1β and IL-6 expression. Additionally, the combination of biochar with AgNPs at 3 µg/mL and AgNPs at 3 µg/mL alone significantly upregulated the anti-inflammatory cytokine TNF-α.

## 3. Discussion

An effective and green production of silver nanoparticles using natural reducing, capping, and stabilizing agents was proposed [29,30]. Recently, there has been a lot of interest in AgNP biosynthesis, and this is the first report employing the fungus isolate *Emericella dentata* to produce AgNPs. The process was optimized for 10 g biomass and 1.0 mM AgNO_3_ at 35 °C and pH 7.0, which took 96 h. It is usually perceived that the size and form of the silver nanoparticles correspond to their absorption peak [31,32]. The reaction mixture’s characteristic brown color was attributed to the Ag nanoparticles’ surface plasmon resonance (SPR) [32]. Although the method of nanoparticle generation is not entirely understood, two putative pathways have been proposed: the quinine shuttle and NADH-dependent nitrate reductase [31,33,34]. It was found that AgNPs have a zeta potential of −19.6 mV, an average hydrodynamic size diameter of approximately 45 nm, and a particle distribution index of 0.231. These characteristics indicate that AgNPs seem to be very durable. Earlier, it was proposed [29,30] that a polydisperse distribution can be inferred if the PDI (DLS) is greater than 0.2. Particles with a high charge tend to have larger Z values because they are more resistant to self-assembly due to their electrostatic resilience. The density distribution in our collection revealed a broad spectrum of shapes. If the relationship between particle size and light scattering was considered, it could be possible that a tiny portion of a bigger component oversees the motion. The sample’s optical properties and the size distribution data obtained from the density distribution were used to construct a relative number that was presented in the number distribution summary [31,32]. Measurements of nanoparticle size distribution zeta indicated that 97.3% of the AgNPs were 37.62 nm in size. Images obtained using scanning transmission electron microscopy (STEM) showed that the particles being investigated were composed of a very large number of extremely minute particles that are smaller than 0.5 µm in size. The analysis of the STEM measurement data was carried out with the assistance of ImageJ, a robust image-processing application that is available for free and under an open-source license [3].

The analysis of one hundred measurements of particle size suggested that biosynthesized AgNPs are round and have an average size of 30 ± 4.3 nm. It was observed that magnification caused the image to blur, making it difficult to determine the structure of the newly synthesized AgNPs. Nanoparticle aggregation into larger composites, the presence of nonconducting carbon stabilizers, and sample charging could also be potential explanations. The nanoparticles seen in the scanning electron microscopy images were spherical and ranged in size from about 10 to 80 nm. STEM images showed a smaller number of AgNPs after drying than were detected by DLS analysis. In fact, smaller than 45 nm is normal for AgNPs, according to several studies [31,33,34]. Porous surfaces were generated because of the volatilization of organic material in DS biochar. Between temperatures of 350 and 550 °C, the pores and deep pathways in biochar became increasingly visible. During the process of increasing the decomposition temperature from 350 to 550 °C, the porosity increased from 31% to 69% [35,36]. Thus, a synergistic potential between AgNPs and biochar was revealed, using 550 °C as the pyrolysis temperature for biochar production.

Silver nanoparticle production was confirmed by the XRD pattern and the presence of distinctive peaks. The detected diffraction peaks confirmed the crystalline nature of produced AgNPs [6,28]. Similar results were reported during the preparation of Ag nanoparticles using edible mushroom extract [35] and geranium leaves [37]. The exhibited peaks in ATR-IR spectra of recently prepared silver nanoparticles demonstrated the presence of several organic functional groups that act as reducing and stabilizing agents on the surfaces of silver nanoparticles [38,39]. The antibacterial potential interactions between AgNPs and biochar were evaluated using the disc diffusion method and MIC utilizing microdilution techniques. AgNPs and biochar together considerably improved the antibacterial activity of AgNPs against all bacterial species. However, none of the studied bacteria were inhibited by biochar alone, which was consistent with previous reports [40]. AgNPs may be helpful in food preservation and packaging due to their effectiveness against *L. monocytogenes* and other foodborne pathogens. AgNPs probably inhibited *B. cereus* and *L. monocytogenes* by interacting with the cell wall [41,42].

Increasing doses of AgNPs were tested on cancer cell lines generated from the A549 cell line as well as fibroblast cells. The A549 cell lines were treated with 3 µg/mL of AgNPs to evaluate the effects of the treatment on gene regulation. When administered to fibroblasts, silver nanoparticles displayed their cytotoxicity at concentrations of 12 µg/mL and above, but their antiproliferative effects on A549 cells were visible at concentrations of 6 µg/mL and higher. On the other hand, it was found that anti-apoptotic genes such as BCL-2 and cyclin D1 encountered severalfold downregulation when they were present in A549 cells that had been treated with 3 µg/mL AgNPs or a combination of 3 µg/mL AgNPs and 50 µg/mL biochar for a period of 24 h. When the A549 cell line was treated with varied concentrations of AgNPs, the effect of the lowest concentration utilized (3 µg/mL of AgNPs) showed that there was a statistically significant rise in caspase 3, the genotype that is associated with pro-apoptotic behavior. Other studies have shown that bioactive substances can influence several anti-apoptotic genes, thereby preventing these genes from performing their immediate protective function against apoptosis and leading to the death of apoptotic cells. This death of apoptotic cells can be attributed to the fact that these genes are unable to perform their immediate protective function against apoptosis [43,44,45].

The administration of AgNPs induced a reduction in the levels of both IL-1β and IL-6, while concurrently leading to an increase in TNF-α levels. It was found that TNF-α was the link between inflammation and cancer and appears to be a critical mediator in this association [46]. TNF-α is a key therapeutic target in several chronic inflammatory disorders [43,47,48]. Such outcomes might be connected, at least in part, to the capacity of biochar’s porous structure to trap and manage the release of such tiny AgNP particles. This was examined when maize plants responded to biochar (2% *w*/*v*) and AgNPs (100–1000 mg/mL) in a hydroponic exposure media [49,50]. It has been observed that surface complexation by biochar might lower Ag+ ions in the growth media by reducing the bioavailability of AgNPs. Combining biochar with AgNPs mitigated their phytotoxicity by decreasing absorption and tissue accumulation of Ag+ ions by a factor of four to eight. Moreover, the reduced oxidative stress seen in plants treated with combined AgNPs and biochar was reflected in the increased activities of antioxidant enzymes [49,50]. Biochar can be used when antimicrobial agents are used to treat antibiotic-resistant bacteria or drugs for chemotherapies aimed at improving cancer treatment along with silver nanoparticles (AgNPs). Our findings provide substantial evidence for the claim that date seed biochar controls AgNP absorption and release. As a result, the biochar becomes more selective for lung cells, assisting in reducing the toxicity and release of AgNPs. The interaction of silver nanoparticles with biochar also leads to their immobilization, which lowers the degree of bioavailability in their surroundings [49]. In conclusion, biochar has the potential to prove useful as a nano-carrier in the fight against antibiotic-resistant pathogens and in the enhancement of treatment.

## 4. Material and Methods

### 4.1. Fungal Strain

The fungus strain W7B employed in this experiment was isolated from soil samples that were taken from the southern Jordanian region of Al-Karak. By using ITS sequencing, the fungus strain’s species was determined (Macrogen, Seoul, Republic of Korea). A sequence similarity analysis using the NCBI database revealed that the ITS sequence of the fungus W7B was 100% identical to that of the *Emericella dentata*, whose accession number is MH032749 according to its identification sheet from Macrogen. The specific bioinformatics report that was supplied by Macrogen for our isolate revealed a 100% match with Emericella dentata, and the report displayed the closest match in comparison to other species as shown in the phylogenic tree.

### 4.2. Bacterial Strains and Reagents

Sigma-Aldrich (Milwaukee, WI 53209, USA) supplied all the chemicals and media used. The organisms used in the antibacterial assay were Gram-positive and Gram-negative. Some of these strains with ATCC numbers were taken as pure cultures purchased from Ben Hayyan Aqaba International Laboratories, Aqaba, Jordan, while others were isolated from UTI patients treated at Karak Government Hospital (KGH) and identified using a VITEK^®^ 2 Compact instrument. This is a highly automated system used for identification and antimicrobial susceptibility testing (bioMerieux, Marcy-l’Étoile, France). The strains of bacteria were sustained by subculturing them on nutrient agar regularly and keeping them at 4 °C before use. All solutions were prepared with deionized water of the highest purity [51].

### 4.3. Raw Materials for Biochar and Processing Conditions

After harvesting the date fruit, date seeds (DSs) were acquired locally. Deionized water was used to clean the air-dried seeds. The seeds were then dried and placed in a desiccator for subsequent analysis and the generation of biochar [36].

### 4.4. Date Seeds’ (DSs) Physicochemical Characteristics

Following 24 h incubation in an oven at 105 °C, the seeds were weighed to measure their wet weight. After drying in a muffle furnace set to 550 °C for two hours, the seeds were heat-treated. Equations (1)–(3) were used to calculate moisture and organic matter [52].

(1) $$\mathrm{Total}\;\mathrm{organic}\;\mathrm{matter}={\;\mathrm{Dry}}_{\mathrm{wt}}-{\;\mathrm{Ash}}_{\mathrm{wt}}$$

where Dry_wt_ is the weight of the biochar after it has been dried at 105 °C for twenty-four hours and Wet_wt_ is the initial biochar weight prior to drying.

(2) $$\mathrm{Total}\;\mathrm{organic}\;\mathrm{matter}\;\left(\%\right)=\left(\frac{\mathrm{organic}\;\mathrm{matter}}{{\mathrm{Dry}}_{\mathrm{wt}}}\right)\times 100\%$$

(3) $$\%\;\mathrm{Moisture}\;\mathrm{content}\;=\left(\frac{\left({\mathrm{Wet}}_{\mathrm{wt}}-{\;\mathrm{Dry}}_{\mathrm{wt}}\right)}{{\mathrm{Dry}}_{\mathrm{wt}}}\right)\times 100$$

The pyrolysis of dehydrated date seeds under the lack of oxygen in a muffle furnace using a sealed porcelain crucible at a temperature of 550 °C for two hours was the method used to make DS biochar. In preparation for its usage in the subsequent trials, the DS biochar was reduced to a particle size of 2 mm by subsequent crushing and screening. Equation (4) was utilized to determine the amount of DS biochar that was produced [53].

(4) $$\mathrm{DS}\;\mathrm{biochar}\;\mathrm{yield}\;\left(\%\right)=\left(\frac{\mathrm{weight}\;\mathrm{of}\;\mathrm{biochar}}{\mathrm{weight}\;\mathrm{of}\;\mathrm{dry}\;\mathrm{sample}}\right)\times 100$$

Transmission electron microscopy (TEM) imaging was used to study the microstructure of DS biochar. The pH level of the DS biochar was studied according to Greenberg et al. [54]. The porosity and water absorption of DS biochar were determined using the Archimedes method in accordance with the accepted method (ASTM C20) as described by Sutcu et al. [55]. This was done to establish the DS biochar’s porosity and water absorption. Equation (5) determined % porosity using the estimated parameters, which are as follows:

(5) $$\mathrm{Porosity}\;\mathrm{percentage}\;\left(\%\right)=\left(\frac{\mathrm{open}\;\mathrm{pore}\;\mathrm{volume}}{\mathrm{total}\;\mathrm{volume}}\right)\times 100\;=\left(\frac{\left({\mathrm{Sat}}_{\mathrm{wt}}-{\;\mathrm{Dry}}_{\mathrm{wt}}\right)}{{\mathrm{Sat}}_{\mathrm{wt}}-{\;\mathrm{Sus}}_{\mathrm{wt}}}\right)\times 100$$

where Sat_wt_ is the weight of the biochar when it has boiled in water for two hours, and Sus_wt_ is the weight of the biochar after it has been submerged in water for twelve hours.

### 4.5. AgNPs Biosynthesis

The technique previously mentioned by Gurunathan et al. was used to examine the fungal strain’s capacity to produce AgNPs [56]. One mL of approximately 10^4^ spore suspension of the fungal isolate *Emericella dentata* was first grown aerobically at 30 °C for 96 h in broth medium (pH 7.0) containing 1.0% (*w*/*v*) glucose, 1.0% (*w*/*v*) yeast extract, and 0.5% (*w*/*v*) NaCl. Filtration was used to collect the fungal biomass, which was completely washed with sterilized distilled water before being resuspended in 100 mL of buffered, sterilized, and deionized water (pH 7.0) and incubated for 72 h in an orbital shaker at 30 °C and 150 rpm. By adding another filtration step, the liquid component of the biomass–deionized water suspension was obtained. Silver nitrate was then added to the filtrate that had been collected. The produced mixture was incubated at a controlled temperature (30 °C) in a shaker incubator (150 rpm) in the dark until a change from a light yellow to a dark brown color was evident.

#### 4.5.1. AgNPs Characterization

Using a UV-1800 spectrophotometer, we measured the absorbance of the resulting brown color, which indicated the synthesis of AgNPs. The reaction solution was centrifuged at 15,000 rpm for 20 min on several occasions to remove the bio-formed silver nanoparticles (MIKRO 200 R, Hettich, Westphalia, Germany). After being washed in deionized water, the nanoparticles were resuspended. Once the silver nanoparticle pellet was recovered, it was dried in a vacuum (VWR 1410 Vacuum Oven, Radnor, PA, USA). TEM images of the manufactured AgNPs’ size distribution, shape, and form were captured using an FEI Versa 3D Dual Beam equipment (FEI, Malvern, PA, USA). MAXima-X XRD-7000 was used to determine the crystalline nature of the synthesized AgNPs. Proteins and other molecular structures were identified using a Bruker Alpha FTIR spectrometer as potential factors in AgNP stability [6,57,58,59].

#### 4.5.2. The Size Distribution of the Particles and Their Zeta Potential

The Zeta-sizer Nano ZS90 was utilized so that both the particle size of the AgNPs as well as their zeta potential could be determined (Malvern Instruments, Malvern, UK). The investigation was conducted at 25 °C and a dispersion angle of 90 degrees utilizing samples of varying intensities that had been prepared with deionized water and distilled water, respectively.

### 4.6. The Antibacterial Activity of Silver Nanoparticles, Biochar, and Their Combination

The biosynthesized AgNPs, biochar, and their combination were tested against Gram-positive bacteria such as *S. epidermidis*, *S. aureus*, *Bacillus cereus* (ATCC 11778), *Listeria monocytogene* (ATCC 7644) as well as Gram-negative bacteria such as *Escherichia coli* 0157:H7 (ATCC 43888), *E. coli*, *P. aeruginosa*, *P. aeruginosa* (ATCC 10145), *Salmonella Typhi* (ATCC 14028), and *E. coli* (ATCC 25922). All the ATCC bacterial strains were supplied by Ben Hayyan–Aqaba International Laboratories, Jordan, and were maintained on MH agar until use. Using the Kirby–Bauer disk diffusion susceptibility test technique, the antibacterial activity of AgNPs, biochar, and their combination against the previously described pathogenic bacteria was assessed [60]. Using a sterile cotton swab, the bacterial strains were dispersed on Mueller–Hinton agar (MHA) (Merck, Darmstadt, Germany). The stock solution for AgNPs, biochar, and their combination were prepared as follows: 1 mg/mL AgNP solution (AgNPs-S), and 1 mg/mL biochar solution (BS) which was kept for 24 h at 25 °C while being shaken. The phosphate-buffered water used as the aqueous solution had a pH of 7.4 (Invitrogen, Waltham, MA, USA). AgNPs-S+BS mixture was made by combining 100 µL of AgNPs-S and 100 µL of BS solutions, and both were vortexed multiple times before use. To obtain 10 µg/disc of AgNPs and 10 µg/disc of biochar, as well as a combined 5 µg/disc of biochar and 5 µg/disc of AgNPs, 10 µL of AgNPs-S, BS, and AgNPs-S+BS were then poured onto each of the sterile 6 mm discs that had been placed on the plate. A center disc, without AgNPs, biochar, or their combination was maintained as control. The plates were then incubated for 24 h in a 37 °C incubator. The presence of growth-inhibitory zones was regularly checked after incubating the plates. The size of the zones of inhibition (ZOIs) was reported after being measured with a ruler. Each test was repeated three times [61,62,63].

#### 4.6.1. MIC Determination

Using the broth micro-dilution method described by the Clinical and Laboratory Standards Institute (Wayne and Clinical and Laboratory Standards Institute 2012). The MIC of AgNPs, which is defined as the lowest concentration of AgNPs at which there is no detectable bacterial growth, was determined. The test was performed in triplicate using the nutrient broth for bacterial growth on 96-well microtiter plates. Each well of the microtitre plate was kept at a final bacterial concentration of 5 × 10^5^ CFU mL^−1^. Several concentrations of AgNPs, ranging from 0.016 to 1024 µg/mL, were examined for their effects on various bacteria. The growth of each of these different bacteria was not inhibited by biochar alone using multiple concentrations. In fact, for all the synergistic testing with varied AgNP concentrations, a fixed biochar concentration of 50 µg/mL was used. Two controls were utilized in the experiment: a positive control, which was a broth that had been combined with bacterial inoculum, and a negative control, which was a broth that was sterile and had not been inoculated. The bacterial microtitre plates were kept in an incubator for a duration of 24 h at a temperature of 37 °C. Then, an estimate of the MIC values was determined by a manual calculation [64].

#### 4.6.2. Cancer Cell Lines’ Culture

Human lung cancer cell line A549 (ATCC CCL-185) and human periodontal ligament fibroblast cell line (PDL) were employed. The human lung cancer cell line A549 (epithelial cell, lung; derived from the metastatic site; CCL-185™) was obtained from the University of Jordan, Amman, Jordan. Human periodontal ligament fibroblasts (PDL) were isolated from the ligament that fastens the molars to the jawbone. Both cell lines were propagated in DMEM with 10% FBS, 10 mM HEPES buffers, 100 µg/L l-glutamine, 50 µg/L gentamicin, 100 µg/L penicillin, and 100 mg/L streptomycin in a humidified 5% CO_2_ incubator at 37 °C.

#### 4.6.3. Cell Harvesting and Counting

First, all the cells were rinsed in flasks measuring 75 cm^2^ with 3–5 mL of phosphate buffer saline (PBS). Next, 1–2 mL of trypsin was added to each flask, and the process was repeated until the cells were able to detach from one another. After the fresh medium was added to each cell line in an amount that was comparable, the mixture was pipetted thoroughly to dislodge any clumps and create a single-cell suspension that was consistent throughout. For each individual cell line, the frequency and ratio of cell propagation were uniquely characterized. After attaining the necessary number of cells, the cells were multiplied through propagation once every two to three days. After combining 25 µL of the collected cell suspension with 100 µL of a trypan blue dye solution containing 4% *w*/*v* of trypan blue, the cells were then counted by moving the cell suspension to the edges of a hemacytometer slide [65].

#### 4.6.4. Cytotoxicity Assay

Both the biochar and the AgNPs were tested separately (3–200 µg/mL) as well as together for their potential to be cytotoxic to cancer cells. Untreated cells were used as control. The viability of the cells present in the culture was utilized as the basis for cytotoxicity studies. After the cells had been plated into 96-well plates with a density of 1 × 10^4^ cells per well and cultured for 24 h at 37 °C in DMEM, the cells were treated for an additional 48 h in DMEM containing various doses of biochar and AgNPs. The MTT assay was then carried out in the following manner: the media were first discarded, and the cells in each well were then incubated at 37 °C for four hours with 20 µL of an MTT solution containing 5 mg/mL of the substance. The MTT solution was then discarded and 200 µL of dimethyl sulfoxide (DMSO) was added to dissolve the insoluble formazan crystals. Optical density readings were taken at 570 and 630 nm (data were collected from three independent wells). Primary cell cultures of human periodontal ligament fibroblasts (PDL) and human lung cancer (cell lines of A549 ATCC CCL-185) were utilized for the verification of selective cytotoxicity with the goal of obtaining the lowest antiproliferative IC50 values. All experiments were carried out in triplicates to ensure accuracy, and the IC50 antiproliferative activities were presented as the mean values with standard deviation (n = 3) [65,66].

#### 4.6.5. Gene Expression Level Assay

Cancer cells were grown in 6-well plates with 1 µg/mL at a density of 1 × 10^6^ cells per well [67]. The changes in fold expression of IL-6, IL-1β, TNF-α, Cyclin D1, BCL-2, and caspase-3 genes were measured after 24 h of incubation with the chosen concentrations of biochar and AgNPs [68].

#### 4.6.6. Ribonucleic Acid (RNA) Extraction and Analysis

RNeasy Mini kit was employed to collect total RNA (QIAGEN, Germantown, MD, USA). Cell pellets were taken out of the −80 °C freezer, defrosted on ice, and then re-suspended in 500 μL lysis solution containing 2-mercaptoethanol. To eliminate cellular debris, an equal volume of 500 μL of 70% ethanol solution was added to the filtering lysate and carefully vortexed. The cell lysates were then passed to the RNeasy Mini spin columns and spun at 10,000 revolutions per minute for 15 s. A binding column contained the whole RNA that had been trapped. The collection tubes were brought back to a binder column after the flow-through liquids were discarded. The tubes were subjected to 3 wash phases. A fresh collecting tube was then used to hold the binding columns. To extract the RNA, 50 μL of RNase-free water was added straight away to the spin columns’ membranes and centrifuged at 10,000 rpm for one minute. Refined RNA was promptly collected and stored at −80 °C. Using Nanodrop ND-1000 spectrophotometers, the recovered total RNA’s concentration and purity were assessed (Thermo Scientific, Wilmington, DE, USA). The optical densities at 260 nm and 280 nm were calculated. For most of the RNA-isolated specimens, the proportion (A260/A280) was between 1.8 and 2.1.

#### 4.6.7. Complementary Deoxyribonucleic Acid (cDNA) Synthesis

cDNA was created utilizing a reverse transcription technique (Applied Biosystem, Waltham, MA, USA). An amount of 2 μg of total RNA and 1 μL of oligo-deoxythymidine primers were added to a microcentrifuge tube and heated on a thermocycler C1000 for five minutes at 65 °C (Bio-Rad, Hercules, CA, USA). The tubes were immediately centrifuged, after which they were placed in ice. The accompanying chemicals were used to create the 20 μL reaction solution: 1.4 μL of 25 mM MgCl_2_ and 4 μL of a combination of 10 mM dNTPs, avian myeloblastosis virus reverse transcriptase (1 μL), recombinant RNasin^®^ ribonuclease inhibitor (1 μL), Shirley, NY 11967, USA, and reverse-transcribed 10× buffers (2 μL) (AMV-RT). The following were the temperature requirements of cDNA synthesis: The microcentrifuge tubes were incubated for 30 min at 37 °C. The specimens were heated for five minutes at 95 °C as part of the denatured protein phase. Following a 5 min incubation period at 4 °C, microcentrifuge tubes were stored at −80 °C for later examination. Nanodrop ND-1000 spectrophotometers were used to measure the amount and quality of cDNA (Thermo Scientific, Wilmington, DE, USA). The optical densities at 260 nm and 280 nm were calculated, and for most of the cDNA retrieved specimens, the proportion (A260/A280) was between 1.6 and 1.8.

#### 4.6.8. Relative Quantitative RT-PCR Analysis

Fast SYBR green kappa master mixes were used for relative quantitative assessments of the mRNA expression rates of the investigated enzyme (Biosystem, Foster City, CA, USA). In each PCR reaction, 200 nM of the forward primers and 200 nM of the reverse primers were mixed with a mixture of 1× concentration KAPA SYBR greens fast master solution, 1–2 μL of cDNA templates, and adjusted to a final volume of 20 μL. Table 3 contains a list of the primer combination sequences that were employed. The IQ5 multicolor real-time PCR detection system (Bio-Rad, USA) was used to carry out the PCR amplifications. Every process ended with melting curves that ranged from 70 to 95 °C. To normalize the expressions of the examined genes, glyceraldehyde-3-phosphate dehydrogenase (GAPDH) was employed as an internal reference gene. The efficiency of the PCR reaction was evaluated using the calibrating curves method for comparable quantitative measurements. The Pffafl technique (Equation (6)) was used to calculate the mRNA expression statistics [69].

(6) $$\mathrm{RQ}=\frac{{\left(E_{\mathrm{target}}\right)}^{\Delta \mathrm{Ct}\left(\mathrm{target}\right)}}{{\left(E_{\mathrm{reference}}\right)}^{\Delta \mathrm{Ct}\left(\mathrm{reference}\right)}}$$

where,

∆Ct(target) = Ct (target gene in calibration) − Ct (target gene in test);

∆Ct(reference) = Ct (reference gene in calibration) − Ct (reference gene in test).

The amplification efficiency was calculated using Equation (7):

(7) $$E=10^{-1/\mathrm{slope}}$$

In one instance, the effectiveness was 2 (the effectiveness of both the targets and references genes is equal to 2), and the slopes were −3.32 if the number of references and targeted DNA areas are doubling each cycle, which is calculable assuming (Equation (8)):

(8) RQ=2ΔCt(target)2ΔCt(reference) =2[ΔCt(target)−ΔCt(reference) ]=2[(Ct(target,calibrator)−Ct(target,test)]−[(Ct(reference,calibrator)−Ct(reference,test)] =2−[(Ct(target,test)−Ct(target,calibrator)]−[(Ct(reference,test)−Ct(reference,calibrator)] =2−ΔΔCt 

Our results were obtained from three independently performed experiments.

### 4.7. Statistical Analysis

A non-parametric Mann–Whitney test was employed to investigate any significant group differences. Additionally, Dunnett’s post hoc test was employed after a one-way analysis of variance (ANOVA) for data that were regularly distributed. SPSS version 22 was used to analyze the data (SPSS, Inc., Chicago, IL, USA). The remaining findings are displayed as the means and standard deviations (SD) of 3–4 separate studies. Dunnett’s post hoc test was used to examine the statistical differences between the control and various treatment groups after performing an ANOVA on GraphPad Prism. A *p*-value of less than 0.05 was regarded as statistically significant for all statistical analyses. A statistical difference was deemed to be very significant if the *p*-value was <0.001.

## 5. Conclusions

In conclusion, this is the first investigation that uses AgNPs and biochar in combination against pathogenic bacteria and lung cancer cells. Although biochar by itself exhibited no antibacterial action, the combination of the two possessed synergistic antibacterial activity (14.5–21.5 mm inhibitory zones). The A549 (ATCC CCL-185) and fibroblast cell lines used in this work were susceptible to AgNPs alone, and the effect was more potent when combined with biochar. The present study found that silver nanoparticles produced by fungi and biochar possess cytotoxic properties that can be used in biomedical applications. Our findings showed that biosynthesized AgNPs with biochar in minute quantities could potentially be employed to treat drug-resistant bacteria and lung cancer epithelial cells.

## Figures and Tables

**Table 1:** Some physical and chemical properties of pyrolyzed DS biochar at 550 °C (n = 3); the results were reported as mean ± SD.

DS Biochar Parameters	Pyrolysis Temperature (550 °C)
pH	7.9 ± 0.15
Porosity (%)	73 ± 4.0
Organic matter (%)	98.56 ± 3.95
Biochar yield (%)	25.2 ± 3.2
Water holding capacity (% WHC)	59.7 ± 4.25

**Table 2:** Inhibition zones and MIC values of AgNPs, biochar, and AgNPs + biochar.

	AgNPs		Biochar		AgNPs + Biochar	
Micro-Organism	Inhibition Zone (mm)	MIC µg/mL	Inhibition Zone (mm)	MIC µg/mL	Inhibition Zone (mm)	MIC µg/mL
*B. cereus* (ATCC 11778)	20.5 ± 0.5	6.38	0.0	Nd	24.5 ± 0.5	2.13
*L. monocytogene* (ATCC 7644)	18.5 ± 0.5	6.38	0.0	Nd	20.5 ± 0.0	2.13
*P. aeruginosa* *	12.5 ± 0.5	19.15	0.0	Nd	14.5 ± 0.4	6.38
*S. typhi* (ATCC 14028)	15.5 ± 0.5	6.38	0.0	Nd	19.5 ± 0.6	2.13
*S. aureus* *	18.5 ± 0.5	6.38	0.0	Nd	21.5 ± 0.5	2.13
*E. coli* *	14.0 ± 0.0	19.15	0.0	Nd	16.5 ± 0.0	6.38
*P. aeruginosa* ATCC 10145 *	12.3 ± 0.0	19.15	0.0	Nd	16.5 ± 0.5	6.38
*E. coli* ATCC 25922 *	13.5 ± 0.6	19.15	0.0	Nd	15.0 ± 0.6	6.38
*S. epidermidis* *	17.5 ± 0.7	6.38	0.0	Nd	19.5 ± 0.0	2.13
*E. coli* 0157:H7 (ATCC 43888)	14.5 ± 0.6	19.15	0.0	Nd	17.0 ± 0.6	6.38

Nd: not detected. The bacterial strains marked with an asterisk are presented in the table for comparison and have been previously studied [27] as well.

**Table 3:** List of primer sequences used for the selected genes.

Gene Name or Symbol	Primer Sequence
*Cyclin D1*	Forward: 5′-ACC TGA GGA GCC CCA ACA A-3′ Reverse: 5′-TCT GCT CCT GGC AGG CC-3′
IL-6	Forward: 5′-GGTACATCCTCGACGGCATCT-3′; Reverse: 5′-GT GCCTCTTTGCTGCTTTCAC-3′
IL-1β	Forward: 5′-ATGGCAACTGTTCCTGAACTCAAC-3′ Reverse: 5′-CAGGACAGGTATAGATTCTTTCCTTT-3′
*TNF-α*	Forward: 5′-ATGAGCACAGAAAGCATGATCC-3′ Reverse: 5′-TCACAGAGCAATGACTCCAAAGTAG-3′
BCL-2	Forward: 5′-AAG CCG GCG ACG ACT TCT-3′ Reverse: 5′-GGT GCC GGT TCA GGT ACT-3′
caspase 3	Forward: 5′-AGCAAACCTCAGGGAAACATT-3′, Reverse: 5′-CTCAGAAGCACACAAACAAAACT-3′

## Data Availability

Not applicable.

## Abbreviations

NPs: nanoparticles; AFM: atomic force microscopy; SEM: scanning electron microscopy; TEM: transmission electron microscopy; XPS: X-ray photoelectron spectroscopy; AgNPs: silver nanoparticles; DS: date seed; Fcc: face-centered cells; MIC: minimal inhibitory concentration; SPR: surface plasmon resonance; STEM: scanning transmission electron microscopy; XRD: X-ray diffraction; BS: biochar solution; ZOIs: zones of inhibition; PBS: phosphate buffer saline; DMSO: dimethyl sulfoxide; GAPDH: glyceraldehyde-3-phosphates dehydrogenases; ANOVA: one-way analysis of variance; SD: standard deviation.
