# Correction: Abu Hajleh et al. Synergistic Effects of AgNPs and Biochar: A Potential Combination for Combating Lung Cancer and Pathogenic Bacteria. *Molecules* 2023, *28*, 4757

**DOI:** 10.3390/molecules31020266

**Published:** 2026-01-13

**Authors:** Maha N. Abu Hajleh, Muhamad Al-limoun, Amjad Al-Tarawneh, Tahani J. Hijazin, Moath Alqaraleh, Khaled Khleifat, Osama Y. Al-Madanat, Yaseen Al Qaisi, Ahmad AlSarayreh, Ali Al-Samydai, Haitham Qaralleh, Emad A. S. Al-Dujaili

**Affiliations:** 1Department of Cosmetic Science, Pharmacological and Diagnostic Research Centre, Faculty of Allied Medical Sciences, Al-Ahliyya Amman University, Amman 19328, Jordan; m.abuhajleh@ammanu.edu.jo; 2Department of Biological Sciences, Faculty of Science, Mutah University, P.O. Box 7, Mutah 61710, Jordan; moallimoun@mutah.edu.jo (M.A.-l.); thijazeen@yahoo.com (T.J.H.); yastq@yahoo.com (Y.A.Q.); ahmsar@mutah.edu.jo (A.A.); 3Prince Faisal Center for Dead Sea, Environmental and Energy Research, Mutah University, Al-Karak 61710, Jordan; amjtar@hotmail.com; 4Pharmacological and Diagnostic Research Center (PDRC), Faculty of Pharmacy, Al-Ahliyya Amman University, Amman 19328, Jordan; alqaralehmoath@yahoo.com (M.A.); a.alsamydai@ammanu.edu.jo (A.A.-S.); 5Department of Medical Analysis, Faculty of Science, Mutah University, Al-Karak 61710, Jordan; haitham@mutah.edu.jo; 6Department of Chemistry, Faculty of Science, Mutah University, Al-Karak 61710, Jordan; madanat@mutah.edu.jo; 7Centre for Cardiovascular Science, Queen’s Medical Research Institute, University of Edinburgh, Edinburgh EH8 9YL, UK

## Missing Citation

In the original publication [1], the citation of reference 27 [2] is missing in Figures 2 and 3, Table 2, and Section 2.1. The omission of this citation was an unintentional oversight. The correct captions of Figures 2 and 3, and the footnote of Table 2 appear below. The citation has now been inserted.

**Table 2.** Inhibition zones and MIC values of AgNPs, biochar, and AgNPs + biochar.Nd: not detected. The bacterial strains marked with an asterisk are presented in the table for comparison and have been previously studied [27] as well.

## Error in Figures

For easier reading, a clearer version of Figure 2 has been updated. In the original publication [1], the authors had generated Figure 3c by removing noisy parts from the original spectrum. The unmanipulated authentic spectrum appears in the new Figure 3c below.

**Figure 2 molecules-31-00266-f002:**
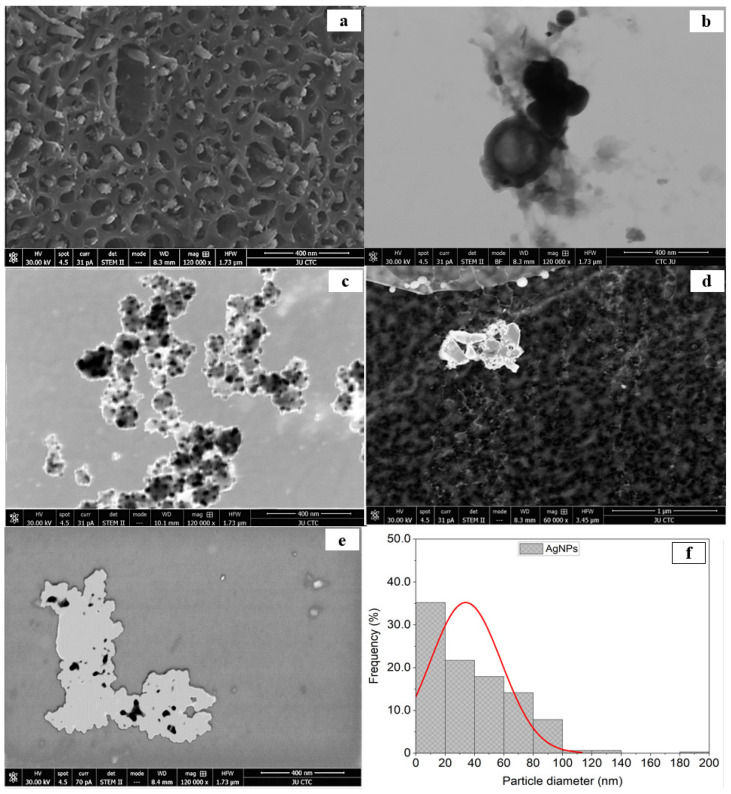
(**a**) STEM micrographs of 400 nm images of biochar, (**b**) STEM micrographs of 400 nm images of biochar [27], (**c**) 400 nm image of silver nanoparticles synthesized by the reaction of 1.0 mM silver nitrate with Emericella dentata filtrate, (**d**) 1000 nm image of combined biochar and AgNPs, (**e**) 400 nm combination of AgNPs and biochar, and (**f**) % frequency of AgNPs size distribution. (Figure 2b shows the identical detail as Figure 2a in [27] and is shown here for easier comparison).

**Figure 3 molecules-31-00266-f003:**
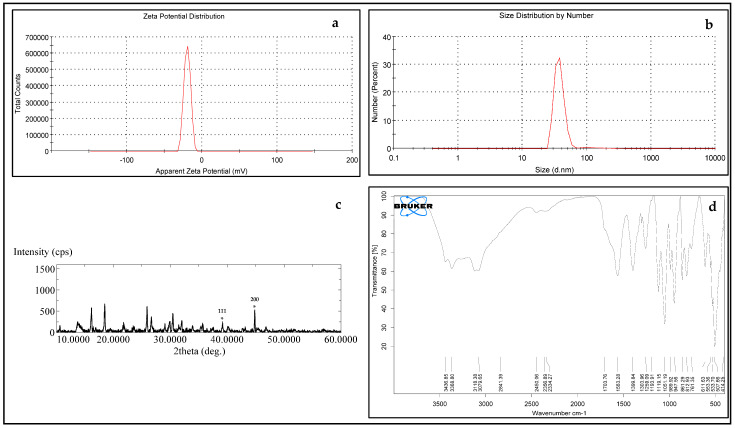
(**a**) Zeta potential distribution of silver nanoparticles [27]; (**b**) the size distribution by the intensity of AgNPs [27]; (**c**) XRD analysis of silver nanoparticles biologically prepared by Emericella dentata filtrate (* assign the reflection planes of the face-centered cubic structure of silver) [27]; and (**d**) ATR-IR of silver nanoparticles biologically prepared by Emericella dentata filtrate. (Figure 3a–c were taken from Figures 1A,B, and 3 in our previous published work [27] and are repeated here for easier comparison).

## Text Correction

There was an error in the original publication about SEM and STEM. Corrections have been made to the fourth sentence in the abstract, the third paragraph in Section 2.1, the last sentence in the first paragraph, and the fifth sentence in the second paragraph in Section 3.

## References

Reference 27 [2] has been added. With this correction, the order of some references has been adjusted accordingly.

The authors state that the scientific conclusions are unaffected. This correction was approved by the Academic Editor. The original publication has also been updated.
